# Even one star at A level could be "too little, too late" for medical student selection

**DOI:** 10.1186/1472-6920-8-16

**Published:** 2008-04-07

**Authors:** Chris McManus, Katherine Woolf, Jane E Dacre

**Affiliations:** 1 Dept of Psychology, University College London, Gower Street, London WC1E 6BT, UK; 2 Academic Centre for Medical Education (ACME), UCL Division of Medical Education (DoME), 4th Floor, Holborn Union Building, Archway Campus, Highgate Hill, London N19 5LW, UK

## Abstract

**Background:**

More and more medical school applicants in England and Wales are gaining the maximum grade at A level of AAA, and the UK Government has now agreed to pilot the introduction of a new A* grade. This study assessed the likely utility of additional grades of A* or of A**.

**Methods:**

Statistical analysis of university selection data collected by the Universities and Colleges Admissions Service (UCAS), consisting of data from 1,484,650 applicants to UCAS for the years 2003, 2004 and 2005, of whom 23,628 were medical school applicants, and of these 14,510 were medical school entrants from the UK, aged under 21, and with three or four A level results. The main outcome measure was the number of points scored by applicants in their best three A level subjects.

**Results:**

Censored normal distributions showed a good fit to the data using maximum likelihood modelling. If it were the case that A* grades had already been introduced, then at present about 11% of medical school applicants and 18% of entrants would achieve the maximum score of 3 A*s. Projections for the years 2010, 2015 and 2020 suggest that about 26%, 35% and 46% of medical school entrants would have 3 A* grades.

**Conclusion:**

Although A* grades at A level will help in medical student selection, within a decade, a third of medical students will gain maximum grades. While revising the A level system there is a strong argument, as proposed in the Tomlinson Report, for introducing an A** grade.

## Background

Selection of university students, particularly for the most competitive and academically demanding subjects, has become more and more difficult during the past decade and a half, as students have attained higher and higher A level grades.

15% of university applicants with three or more A levels now gain the maximum possible mark of three As, making selection increasingly problematic, particularly in subjects such as medicine, where 43% of applicants and 61% of entrants now gain AAA. The result is that many medical schools are now using tests of general intellectual ability for selection for which until recently there was little evidence of predictive validity (although the assessments are reliable and have some construct validity) [1]. Since submission of the first draft of this paper, BMAT has provided some evidence for predictive validity for BMAT's Section 2 (Scientific Knowledge and Applications) on academic examinations [2].

Currently students must achieve 80–100% on the UMS [uniform mark scale] to achieve an A grade, 70–79 for a B, 60–69 for a C, 50–59 for a D and 40–49 for an E [3]. In March 2007, the then Education Secretary Alan Johnson agreed the Qualifications and Curriculum Authority's recommendations to award an A* grade to "those students who have achieved a grade A at A level and who have achieved at least 90% of their UMS [uniform mark scale]on the aggregation of their A2 units" [4]. This system will be piloted with students starting their A levels in September 2008. In 2004 the Tomlinson Report [5] had not only suggested an A* grade at A level, but also a grade of A**. Here we suggest that a single additional grade of A* will soon be of little help to medical educators, and that a higher grade of A** should be introduced as the system is being revised.

## Methods

UCAS provided data on all 1,484,650 university applicants for the years 2003 to 2005, of whom 528,691 applicants were home applicants aged under 21 who had attained three or four A levels, excluding General Studies (3 A levels, n = 473,781; 4 A levels, n = 54,910). A level scores were calculated on the basis of A = 10, B = 8, C = 6, D = 4 and E = 2, with the highest three of the grades being scored for those with four A levels. Data on medical school applicants were also available for 13,377 medical school applicants in 1996 and 1997 aged under 21 who had taken A levels. Statistical analysis used SPSS 11.5, with maximum likelihood modelling of censored distributions carried out using a purpose-written Excel spreadsheet (see Additional File 1).

## Results

### All applicants

**Figure 1:**
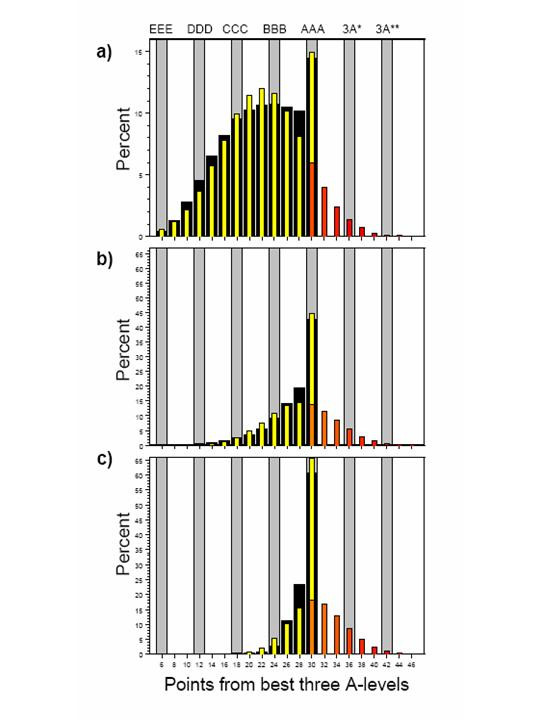
Solid black bars show observed distribution of A level points from best three A level grades, scored as A = 10, B = 18, C = 6, D = 4, E = 2 for a) all UCAS applicants, b) all medical school applicants and c) all medical school entrants. Yellow bars show fitted results of a censored normal distribution. The yellow bar at 30 points (AAA) can be decomposed into the red bars for the fitted, uncensored normal distribution, scored as 12 = A* and 14 = A**. The ordinates of medical school applicants and entrants are on the same scale to allow comparison.

The wide black bars in Figure 1a show the percentage of all UCAS candidates gaining the various grades. There is clearly a ceiling at 30 points (AAA), which is also the modal value, gained by 14.5% of these applicants. Modelling of a normal distribution, censored at 30 points, showed a reasonable fit to the data (yellow bars, mean = 22.1, SD = 6.60). The red bars in Figure 1a show the expected distribution of applicants gaining more than 30 points from 3 A levels, calculated as A* = 12, A** = 14, etc.. If A* were introduced, then at present about 3% of all university applicants would have 36 points or more (3 A*).

### Applicants to Medicine

Figures 1b and 1c show the A level grades of 23,628 applicants and 14,510 acceptances at medical school, 44.9% and 61.9% having AAA (30 points). Fitting a censored normal distribution estimates the true mean (SD) A level points as 28.3 (5.5) for applicants and 30.1 (4.5) for acceptances, with 11% and 18% currently expected to gain 36 or more points (i.e. 3 A*s or better).

### Trend across years

41.6%, 42.5% and 44.1% of medical applicants in 2003, 2004 and 2005 scored 30 points, as compared with 30.6% in 1996 and 1997. 54.9%, 60.3%, and 66.1% of entrants in 2003–5 scored 30 points.

## Discussion

One in seven of all university applicants and nearly a half of medical school applicants currently gain maximum marks at A level, making selection difficult when about 60% of medical applicants are accepted. Estimation of censored distributions however allows prediction of the likely achievement of candidates currently at the ceiling of AAA. Grades A* are scaled similarly to the current A, B, C, D and E and assuming the same is true of A** grades, then only 11% of medical school applicants and 18% of entrants would gain 3 A* grades or better, improving the process of selection.

For whatever reasons, inflation of A level grades continues to occur, with 25.3% of all scripts in 2007 gaining an A grade [6]. The proportion of AAA grades is increasing at about 1.6% per annum in medical applicants, with a higher rate in entrants. Projecting forward on the basis of such figures (see Additional Files 1 and 2 for details) suggests that about 17%, 22% and 29% of applicants and 26%, 35% and 46% of entrants will have 3 A*s by 2010, 2015 and 2020.

Although A levels are used for selection, the justification for that use comes from being valid predictors of outcome in and beyond medical school [1,7]. Validation however is difficult to obtain when most students gain maximum marks (although shown in Additional Files 1 and 3, recent students with grades of AAB, ABB and BBB do still perform less well than those with AAA). The introduction of A** grades would allow the predictive validity of A levels to continue to be assessed properly. It should also be emphasised that validity is not something that can be assumed for all tests for all times. Tests and times change, and validity needs therefore to routinely monitored for any selection method. Predictive validity should also be examined broadly, not merely for examination success, but for other aspects of real-world clinical practice in working doctors.

## Conclusion

While A levels are being revised to include a grade of A* it would therefore be prudent also to include a grade of A**, as Tomlinson suggested ^4^, to allow the system to continue to work effectively in the future.

## Competing interests

The author(s) declare that they have no competing interests.

## Authors' contributions

ICM conceived of the study, analysed the UCAS data and wrote the first draft of the manuscript. KW and JD obtained the UCAS and medical school examinations data. KW analysed the medical school examination data, and contributed to subsequent drafts of the manuscript. All authors read and approved the final manuscript.

## Pre-publication history

The pre-publication history for this paper can be accessed here:



## Supplementary Material

Additional file 1 Supplementary information. Information to supplement the main manuscript, including the estimation of the censored distributions for applicants and entrants to medical school; secular trends across A level results in applicants to medical school; and the predictive validity of A levels. Click here for file

Additional file 2 Supplementary figure 1. The distribution of ALEVUCAS (i.e. UCAS's calculation of the points obtained from the top three best A level grades, including General studies, by students in 1996–7), restricted to those aged 20 or less, and taking three or four A levels. Click here for file

Additional file 3 Supplementary figure 2. Performance of third year UCL medical students in MCQ and OSCE assessments by top three A level points. Click here for file
