# Supplementary material for: Even one star at A level could be "too little, too late" for medical student selection
Source: BMC Med Educ. 2008 Apr 7;8:16. doi: 10.1186/1472-6920-8-16 (PMC2335100; doi:10.1186/1472-6920-8-16)
Supplement: Additional file 1 — Supplementary information. Information to supplement the main manuscript, including the estimation of the censored distributions for applicants and entrants to medical school; secular trends across A level results in applicants to medical school; and the predictive validity of A levels. [file 1472-6920-8-16-S1.doc]

**Supplementary Information**

i) **Estimation of the censored distributions.**

Estimation of the estimated true parameters of a censored normal distribution was carried out using a maximum likelihood procedure implemented within an Excel spreadsheet. Slightly different procedures were used when only data for applicants was available (as in the 1996-7 data), and when applicant and entrant data was available (as in the 2003-5 data).

**Applicant data only**. Expected values for particular true mean and true SD were calculated using cumulative normal distributions calculated with the NORMDIST function for each A level grade from 6 to 30 points, and for hypothetical points values from 30 to 54 (with a step size of 2). Expected and observed values were compared by calculating the log likelihood in the standard way, and SOLVER function in EXCEL used to find the estimated true mean and SD which gave the maximum likelihood value.

**Applicant and entrant data**. Experience with using the procedure described above separately for applicants and entrants sometimes showed the anomalous finding that more applicants than entrants gained high grades. The solution is to estimate the distributions for applicants and entrants simultaneously. As before, the estimated true mean and true SD for applicants was used to calculate an expected grade distribution for applicants. In addition, the probability of being accepted with a particular A level score was modelled using a conventional two-parameter logistic distribution, the two parameters being the score with a 50% likelihood of acceptance (the *b* parameter, equivalent to the intercept in conventional regression or the difficulty parameter in item response theory), and a slope parameter (the *a* parameter, equivalent to the slope in conventional regression, or the discrimination parameter in item response theory). Using the logistic regression, the expected number of individual being accepted or rejected at each A level score was calculated. The log likelihood was then calculated to assess the similarity of the observed and predicted results. It should be noted in doing this that the marginal totals for the number of rejected and accepted applicants was fixed, whereas the marginal totals for the total number of applicants with particular grades was random. SOLVER was then used to find the maximum likelihood estimates of the four free parameters, and from these values were also calculated two other derived parameters, the estimated true mean and true SD for entrants.

Copies of the EXCEL spreadsheets are available on request from the first author.

**ii) Secular trends**

In order to calculate secular trends across A levels in those applying to medical school, it is necessary to have some estimate of the rate of change of A level grades in medical school applicants and entrants.

*2003-2005.* The UCAS data are available for the years 2003, 2004 and 2005, and as the table below shows, the percent of AAA grades, as well as the estimated true means have been increasing during that period, from 28.09 in 2003 to 28.49 in 2005, an increase of 0.20/year. Clearly, though, that time period is short.

*1996-7.* Few data are available for other periods, and the only adequate dataset was that provided by UCAS for the analysis carried out on behalf of CHMS (Council of Heads of Medical Schools) in 1998, which contained data for the years 1996 and 1997 and is in the public domain (see McManus(1998)^1^ ). The 1996-7 dataset is not perfect, but does include UCAS’s own calculation of the three best A level grades obtained (which however includes General Studies), which we call ALEVUCAS, and a specially commissioned measure which was the total A level points obtained excluding General Studies (which we called ALEVG). However the latter variable includes all A levels taken, and hence is higher for those taking four or more A-levels than for those taking three, although the number of A-levels taken is also known. Using these data together then,

1. For ALEVUCAS, the distribution of points, restricted to those aged 20 or less, and taking three or four A levels (as in the 2003-5 analysis) is shown in Additional File 1. The fit is not quite as good as for 2003-5, but is reasonable. 30.6% of applicants gain AAA, as compared with 42-44% in the 2003-5 data. The estimated true mean (SD) for 1996 and 1997 is 26.45, and the estimated true SD is 5.94, the latter being broadly similar to that in the 2003-5 data (5.28-5.64), but is a little higher, as would be expected as fewer individuals are censored. The estimated mean of 26.46 is somewhat lower than the 2003-5 values (28.04 to 28.44).
2. The ALEVUCAS measure contains General Studies, whereas the 2003-5 measures do not. Considering just the 1996 and 1997 applicants taking only three A levels, the correlation of ALEVUCAS with ALEVG is 0.929, suggesting the measures are broadly equivalent, but the *raw* mean for ALEVUCAS of 24.98 is somewhat higher than the *raw* mean for AP which is 24.65, a difference of 0.33. On that basis, the fitted mean described above of 26.46, has been adjusted by taking away 0.33, to give a value of 26.13, which should be broadly comparable to that derived for the 2003-5 data.
3. Taking the period from 1996-7 to 2003-5, the estimated true mean increased from 26.46 to 28.25, a rate of 0.24 per year (based on the 7.5 years from mid-1995-6 to mid 2003-2005). That rate has therefore been used for projected forward from 2005, using the aggregate SD of 5.46 found for 2003-5, in order to estimate the likely proportions of candidates with AAA and other grades in 2010, 2015 and 2020 amongst medical school applicants and entrants.
4. Projecting forward for medical school acceptances is based on the slope parameter (*a*) in the logistic regression being the same across years as the aggregate value for 2003-5 (0.210), with the intercept parameter (*b*)fitted to the same value as the aggregate for 2003-5 (26.32), and increasing at the same rate as the increase in the estimated true mean for applicants. In effect, therefore, the selection process itself remains fixed as in the 2003-5 period; this was necessary as the 1996-97 data cannot readily be used to obtain equivalent figures for acceptances, the data set only showing which individuals had received offers (which was appropriate for the purposes of the study). The projections are shown in the Supplementary table 1.

ii) **Predictive validity of A levels**.

A key assumption of reliance on A levels for medical student selection is that A levels are indeed valid predictors of medical school outcome. Although the literature contains a number of examples, most of these are for entrants admitted before the current rise in A level grades, so that a majority of students are achieving AAA grades. Here we provide an example of validation from two London medical schools.

The graph in Additional File 2 shows the performance of third year medical students from two London medical schools, one in the academic years 2002/3 and 2003/4 and the other in the academic year 2004/5 in similarly structured MCQ (multiple choice questionnaire) and OSCE (objective structured clinical examinations) assessments. Differences in mean and SD between schools and years have been removed by expressing results as z-scores within year and school. Error bars show 95% confidence intervals for the mean. The differences in performance in relation to grade are significant (MCQ: one-way ANOVA: F(3,630)=18.49, p<.001; unweighted linear trend, F(1,630)=25.39, p<.001; non-linear trend F(2,630)=2.32, p=.098; OSCE: one-way ANOVA: F(3,639)=7.31, p<.001; unweighted linear trend, F(1,639)=p<.001; non-linear trend F(2,639)=1.24, p=.290).

**Supplementary references**

[1] McManus, I. C. (1998) "Factors affecting likelihood of applicants being offered a place in medical schools in the United Kingdom in 1996 and 1997: retrospective study", *British Medical Journal*, 317, 1111‑1116.

**Supplementary Table 1:** Actual measured data values are shown in **bold**, whereas estimated values such as estimated true means and SDs are shown in *italics*, and projected values are shown in *italics within brackets*.

|  | ------------------------------------------**Data** --------------------------------------- | | | | | | |  | *------------ Projections ----------* | | |
| --- | --- | --- | --- | --- | --- | --- | --- | --- | --- | --- | --- |
|  | 1996-1997 | 1996 | 1997 | 2003 | 2004 | 2005 | 2003-2005 |  | *2010* | *2015* | *2020* |
| *Per cent with AAA* |  | | | | | | |  |  | | |
| All applicants | **-** | **-** | **-** | **14.0%** | **14.7%** | **14.6%** | **14.5%** |  | *-* | *-* | *-* |
| Medical school applicants | **30.6%** | **29.4%** | **31.7%** | **41.6%** | **42.5%** | **44.1%** | **42.8%** |  | *(55%)* | *(63%)* | *(71%)* |
| Medical school entrants | **-** | **-** | **-** | **54.9%** | **60.3%** | **66.1%** | **60.4%** |  | *(77%)* | *(85%)* | *(90%)* |
| *Estimated percent with 3@A** | ---------------***-Estimated values*** ------------- | | | | | | |  |  | | |
| Medical school applicants | *7.5%* | *7.1%* | *7.9%* | *10.8%* | *10.7%* | *10.8%* | *10.8%* |  | *(17%)* | *(22%)* | *(29%)* |
| Medical school entrants | *-* | *-* | *-* | *15.6%* | *17.3%* | *19.3%* | *17.5%* |  | *(26%)* | *(35%)* | *(46%)* |
| *Estimated percent with 3@A*** |  | | | | | | |  |  | | |
| Medical school applicants | *0.7%* | *0.7%* | *0.7%* | *1.0%* | *1.0%* | *0.9%* | *1.1%* |  | *(2%)* | *(3%)* | *(5%)* |
| Medical school entrants | *-* | *-* | *-* | *1.6%* | *1.6%* | *1.6%* | *1.6%* |  | *(3%)* | *(5%)* | *(8%)* |
| *Parameter estimates* |  | | | | | | |  |  | | |
| Medical applicants |  |  |  |  |  |  |  |  |  |  |  |
| Fitted mean | *26.46^+^* | *25.84^+^* | *26.73^+^* | *28.04* | *28.21* | *28.44* | *28.25* |  | *(29.69)* | *(30.89)* | *(32.09)* |
| Fitted SD | *5.94* | *6.01* | *5.85* | *5.64* | *5.47* | *5.28* | *5.46* |  | *(5.46)* | *(5.46)* | *(5.46)* |
| Logistic slope (a) | *-* | *-* | *-* | *.228* | *.222* | *.223* | *.210* |  | *(.210)* | *(.210)* | *(.210)* |
| Logistic constant (b) | *-* | *-* | *-* | *24.49* | *26.26* | *27.81* | *26.32* |  | *(27.76)* | *(28.96)* | *(30.15)* |
| Medical acceptances |  | | | | | | |  |  | | |
| Fitted mean (derived)^++^ | *-* | *-* | *-* | *30.31* | *30.87* | *31.43* | *30.88* |  | *(32.33)* | *(33.53)* | *(34.72)* |
| Fitted SD (derived)^++^ | *-* | *-* | *-* | *4.65* | *4.45* | *4.24* | *4.48* |  | *(4.50)* | *(4.50)* | *(4.50)* |

^+^ Adjusted for the 97-97 data including General Studies (see text for details).

^++^ Note that the parameters for acceptances are derived from those for applicants and the selection process. There are therefore only four independent parameters being estimate
