# Supplementary material for: Even one star at A level could be "too little, too late" for medical student selection
Source: BMC Med Educ. 2008 Apr 7;8:16. doi: 10.1186/1472-6920-8-16 (PMC2335100; doi:10.1186/1472-6920-8-16)

**Supplementary figure 1**: The distribution of ALEVUCAS (i.e. UCAS’s calculation of the points obtained from the top three best A level grades, including General studies, by students in 1996-7), restricted to those aged 20 or less, and taking three or four A levels.


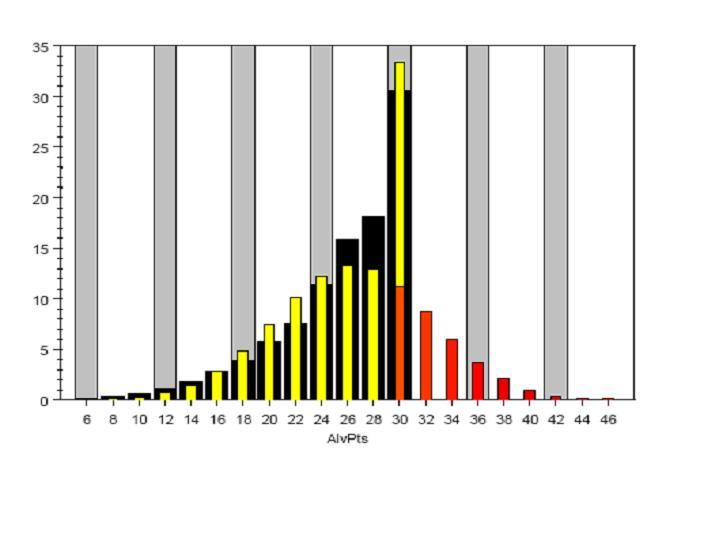

Supplement: Additional file 2 — Supplementary figure 1. The distribution of ALEVUCAS (i.e. UCAS's calculation of the points obtained from the top three best A level grades, including General studies, by students in 1996–7), restricted to those aged 20 or less, and taking three or four A levels. [file 1472-6920-8-16-S2.doc]
