# Supplementary material for: Even one star at A level could be "too little, too late" for medical student selection
Source: BMC Med Educ. 2008 Apr 7;8:16. doi: 10.1186/1472-6920-8-16 (PMC2335100; doi:10.1186/1472-6920-8-16)

**Supplementary figure 2:** Performance of third year UCL medical students in MCQ and OSCE assessments by top three A level points.


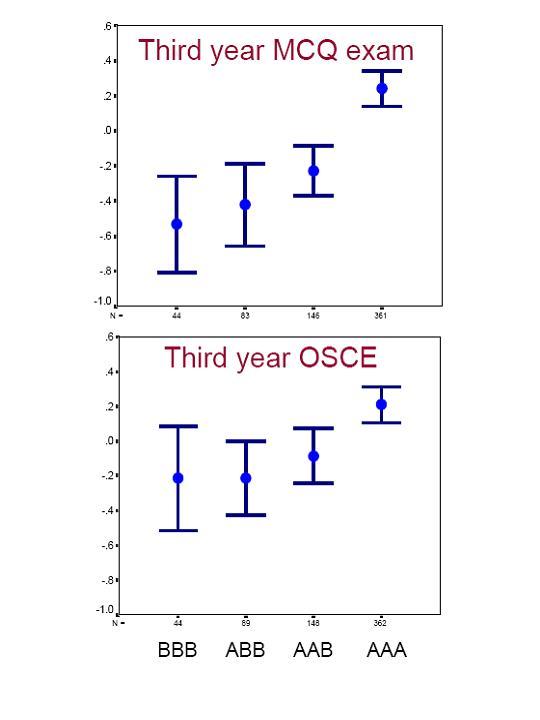

Supplement: Additional file 3 — Supplementary figure 2. Performance of third year UCL medical students in MCQ and OSCE assessments by top three A level points. [file 1472-6920-8-16-S3.doc]
